# Assessing the precision of machine learning for diagnosing pulmonary arterial hypertension: a systematic review and meta-analysis of diagnostic accuracy studies

**DOI:** 10.3389/fcvm.2024.1422327

**Published:** 2024-08-27

**Authors:** Akbar Fadilah, Valerinna Yogibuana Swastika Putri, Imke Maria Del Rosario Puling, Sebastian Emmanuel Willyanto

**Affiliations:** ^1^ Brawijaya Cardiovascular Research Center, Department of Cardiology and Vascular Medicine, Faculty of Medicine, Universitas Brawijaya, Malang, Indonesia; ^2^ Faculty of Medicine, Brawijaya University, Malang, Indonesia

**Keywords:** machine learning, pulmonary arterial hypertension, diagnostic method, area under the curve, area under receiving operator curve

## Abstract

**Introduction:**

Pulmonary arterial hypertension (PAH) is a severe cardiovascular condition characterized by pulmonary vascular remodeling, increased resistance to blood flow, and eventual right heart failure. Right heart catheterization (RHC) is the gold standard diagnostic technique, but due to its invasiveness, it poses risks such as vessel and valve injury. In recent years, machine learning (ML) technologies have offered non-invasive alternatives combined with ML for improving the diagnosis of PAH.

**Objectives:**

The study aimed to evaluate the diagnostic performance of various methods, such as electrocardiography (ECG), echocardiography, blood biomarkers, microRNA, chest x-ray, clinical codes, computed tomography (CT) scan, and magnetic resonance imaging (MRI), combined with ML in diagnosing PAH.

**Methods:**

The outcomes of interest included sensitivity, specificity, area under the curve (AUC), positive likelihood ratio (PLR), negative likelihood ratio (NLR), and diagnostic odds ratio (DOR). This study employed the Quality Assessment of Diagnostic Accuracy Studies-2 (QUADAS-2) tool for quality appraisal and STATA V.12.0 for the meta-analysis.

**Results:**

A comprehensive search across six databases resulted in 26 articles for examination. Twelve articles were categorized as low-risk, nine as moderate-risk, and five as high-risk. The overall diagnostic performance analysis demonstrated significant findings, with sensitivity at 81% (95% CI = 0.76–0.85, *p* < 0.001), specificity at 84% (95% CI = 0.77–0.88, *p* < 0.001), and an AUC of 89% (95% CI = 0.85–0.91). In the subgroup analysis, echocardiography displayed outstanding results, with a sensitivity value of 83% (95% CI = 0.72–0.91), specificity value of 93% (95% CI = 0.89–0.96), PLR value of 12.4 (95% CI = 6.8–22.9), and DOR value of 70 (95% CI = 23–231). ECG demonstrated excellent accuracy performance, with a sensitivity of 82% (95% CI = 0.80–0.84) and a specificity of 82% (95% CI = 0.78–0.84). Moreover, blood biomarkers exhibited the highest NLR value of 0.50 (95% CI = 0.42–0.59).

**Conclusion:**

The implementation of echocardiography and ECG with ML for diagnosing PAH presents a promising alternative to RHC. This approach shows potential, as it achieves excellent diagnostic parameters, offering hope for more accessible and less invasive diagnostic methods.

**Systematic Review Registration:**

PROSPERO (CRD42024496569).

## Introduction

Pulmonary Arterial Hypertension (PAH) is a severe cardiovascular condition marked by increased blood pressure in the pulmonary arteries, resulting in gradual harm and eventual failure of the right side of the heart (1, 2). Traditional diagnostic techniques, particularly right heart catheterization (RHC), have been widely regarded as the most reliable method for evaluating PAH. Although RHC offers precise hemodynamic measurements, its invasive nature presents inherent risks to patients and may hinder prompt diagnosis (3, 4).

The emergence of machine learning (ML) technologies in recent years has brought about a significant change in medical diagnostics, providing non-invasive alternatives that question the existing practices (5). Machine learning techniques utilize computational algorithms to analyze intricate datasets and extract significant patterns, empowering clinicians to make accurate and prompt diagnosis. This paper investigates the potential of non-invasive machine learning (ML) methods to completely transform the diagnosis of pulmonary arterial hypertension (PAH) (6). These methods offer a safer and more patient-friendly alternative to the conventional invasive approaches currently used. The constraints of right heart catheterization (RHC), which encompass the inherent procedural hazards and discomfort, underscore the necessity for pioneering diagnostic instruments. Non-invasive machine learning methodologies, such as advanced analysis of images, processing of signals, and recognition of patterns, offer a chance to overcome these difficulties. ML models can achieve high accuracy in identifying PAH by utilizing data from diverse sources such as medical imaging, patient history, and physiological parameters to detect subtle patterns (7).

This paper examines the current state of diagnosing pulmonary arterial hypertension (PAH), highlighting the limitations of invasive methods, and highlighting the potential of machine learning (ML) as a revolutionary force. We explore the different non-invasive machine learning methodologies used in PAH research, examining their advantages, constraints, and future potential. In addition, we emphasize the ethical and practical factors related to the implementation of machine learning in clinical practice, guaranteeing a thorough comprehension of the consequences for patient care. This paper aims to shed light on the path toward a new era in diagnosing pulmonary arterial hypertension (PAH) by exploring the intersection of machine learning and cardiovascular medicine. By adopting non-invasive machine learning techniques, our goal is to not only question the traditional approach of invasive procedures but also reshape the field of cardiovascular diagnostics. This will ultimately improve patient outcomes and enhance the overall management of pulmonary arterial hypertension.

## Methods

This meta-analysis was conducted based on the Preferred Reporting Items for Systematic Reviews and Meta-Analyses (PRISMA) statement guidelines (8). This study was registered in PROSPERO with registration number CRD42024496569.

### Search strategy

The literature search was carried out on six databases, namely PubMed, ScienceDirect, ProQuest, Taylor & Francis, Embase, and EBSCO until December 2023. The literature search was carried out with keywords using Boolean operators: (“machine learning” OR “deep learning” OR “artificial intelligence”) AND (“pulmonary hypertension”) AND (“sensitivity” OR “specificity” OR “AUC” OR “ROC” OR “AUROC” OR “PPV” OR “NPV” OR “TN” OR “FN” OR “TP” OR “FP”).

### Study eligibility criteria

Study Inclusion and exclusion criteria were determined before the literature search to make the results specific and homogenous. The inclusion criteria were (1) data available or accessible in English language, (2) studies that involve patients with PAH as their sample, (3) studies that use right heart catheterization (RHC) for making PAH diagnosis, and (4) studies that include at least one diagnostic data to be analyzed in this study, namely: true/false negative value, true/false positive value, specificity, sensitivity, area under curve (AUC), area under receiver operating characteristic (AUROC) curve, positive predictive value (PPV), and negative predictive value (NPV). The exclusion criteria were (1) non-human sampling studies and (2) irretrievable articles or articles with incompatible language. Using these inclusion and exclusion criteria, four authors independently assessed the eligibility of the papers, and any disagreements were resolved through discussion.

### Outcome measures

The primary outcome measures of this study are the sensitivity and specificity of overall diagnostic performance. The secondary outcomes are the sensitivity and specificity of subgroup diagnostic performance, namely ECG, blood biomarkers, echocardiography, miRNA, and other subgroups (chest x-ray, clinical code, MRI, and CT scan). All authors independently extracted the outcomes from the included papers to be used for quantitative analysis and any disagreements were resolved through discussion.

### Quality assessment and statistical analysis

The risk of bias for each study will be independently assessed by all reviewers as low, moderate, or high using the tool, Quality Assessment of Diagnostic Accuracy Studies 2 (QUADAS-2) (9). Diagnostic meta-analysis will be performed using STATA V.12.0 (10). The software will be used to test the heterogeneity, and the pooled sensitivity, specificity, positive likelihood ratio, negative likelihood ratio, diagnostic odds ratio, and other effect sizes to generate the summary receiver operating characteristic (SROC) curve for comprehensive evaluation. If high heterogeneity is found, meta-regression analysis will be done to explore the source of heterogeneity alongside subgroup analysis. The competing diagnostic tests will be ranked by their superiority index. Begg's funnel plots will also be used to assess publication bias in the meta-analysis of the diagnostic studies.

## RESULTS

### Study selection and identification

**Figure 1:**
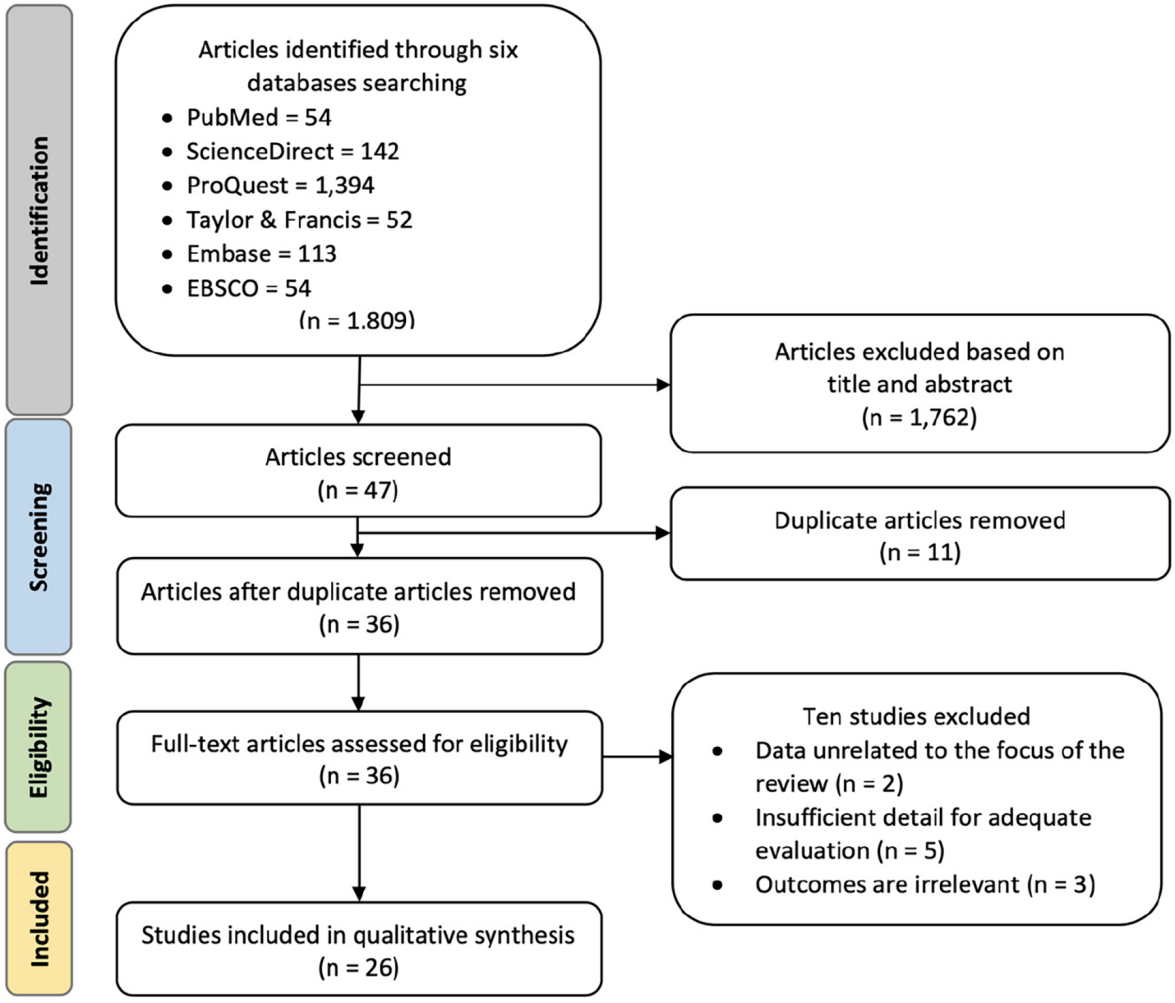
Preferred reporting items for systematic reviews and meta-analyses (PRISMA) flowchart for study identification and selection. The original database search resulted in 1,809 studies from six databases searched, namely PubMed, ScienceDirect, ProQuest, Taylor & Francis, Embase, and EBSCO. Through title and abstract screening, 1,762 articles were removed, and 47 articles were screened for duplication. Duplicate screening resulted in 11 removed articles. Thirty-six articles were further assessed for eligibility and ten articles were removed due to irrelevant data, evaluation, or outcomes. The final step resulted in 26 clinical trials included in the qualitative synthesis.

After removing duplicate studies and screening abstracts, a thorough assessment was conducted on a total of 36 clinical trial studies. Ultimately, 26 clinical trials were selected for inclusion in the meta-analysis, as depicted in Figure 1. Two studies were excluded because their data was unrelated to the focus of this study, five were excluded due to insufficient details for a comprehensive evaluation, and three were excluded as their outcomes were irrelevant to the aim of this study. The selected studies underwent evaluation for quality and were extracted for subsequent analysis using statistical methods.

### Demography and clinical characteristics of the included studies

The demography and clinical characteristics of 26 included studies were examined and listed in Supplementary Table S1.

### Quality appraisal

**Figure 2:**
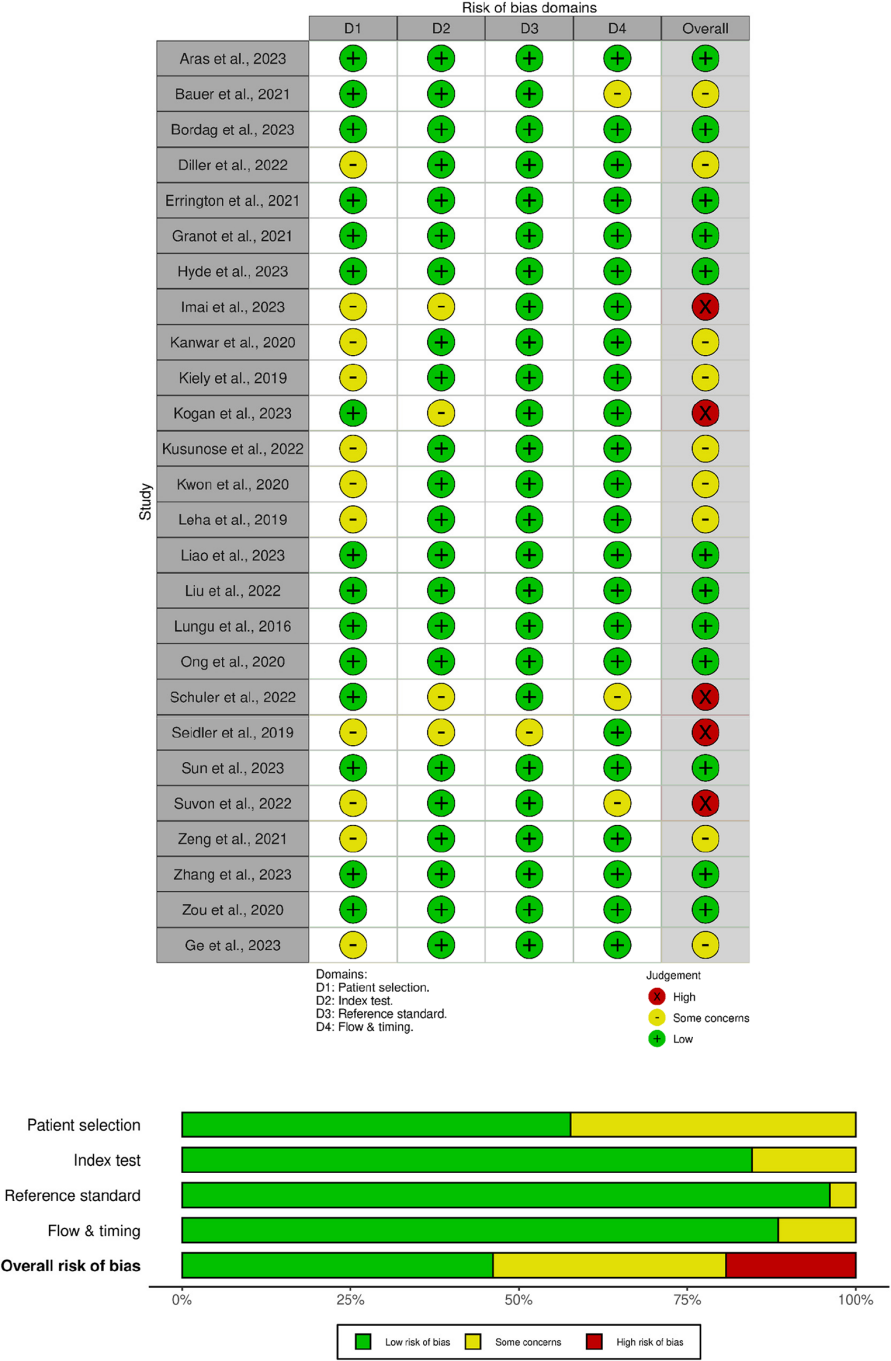
Risk of bias summary using the QUADAS-2 tool for diagnostic studies. The green region represents studies with a low risk of bias, the yellow region shows studies with a moderate risk of bias, and the red region shows studies with a high risk of bias.

The final clinical trials included in the analysis underwent a comprehensive quality evaluation using the QUADAS-2 tool (Figure 2). The assessment revealed that 12 of the studies had a low risk of bias across the four domains evaluated. However, there were nine studies with a moderate risk of bias and five with a high risk of bias. Notably, studies conducted by Diller et al., 2022; Imai et al., 2023; Kanwar et al., 2020; Kiely et al., 2019; Kusunose et al., 2022; Kwon et al., 2020; Leha et al., 2019; Seidler et al., 2019; Suvon et al., 2022; and Zeng et al., 2021 (6, 7, 11–18) did not implement any randomization process in their methodology, thus they were considered to have moderate regarding bias in the patient selection domain. Furthermore, studies conducted by Imai et al., 2023; Kogan et al., 2023; Schuler et al., 2022; and Seidler et al., 2019 (12, 16, 19, 20) had unclear pre-specified thresholds in their diagnostic method standards, leading to a moderate risk of bias in the index test domain. Additionally, the study by Seidler et al., 2019 (16) had an incomplete reference standard for interpreting results, resulting in a moderate risk of bias in the reference standard domain. Lastly, studies conducted by Bauer et al., 2021; Schuler et al., 2022; and Suvon et al., 2022 (17, 20, 21) had the notable loss to follow-up samples, which raised a moderate risk of bias in the flow & timing domain.

### Overall diagnostic performance analysis

A total of twenty-six studies were included in the meta-analysis for the overall diagnostic method, consisting of 6 studies for ECG, 6 studies for echocardiography, 4 studies for blood biomarkers, 5 studies for miRNA, and 6 studies for other diagnostic methods, including chest x-ray, clinical code, MRI, and CT scan. These parameters are analyzed with various thresholds with the number of studies included and cases, their combined sensitivities, and specificities shown in Table 1.

**Table 1:** Subgroup analysis of diagnostic method in machine learning study for pulmonary hypertension.

Diagnostic Method	Sensitivity (95% CI)	Specificity (95% CI)	Positive likelihood ratio (PLR)	Negative likelihood ratio (*N*LR)	Diagnostic odds ratio (DOR)	Sensitivity				Specificity			
						Q	df	p	*I* ^2^	Q	df	p	I^2^
ECG	0.82 [0.80–0.84]	0.82 [0.78–0.84]	4.5 [3.7–5.4]	0.22 [0.19–0.25]	21 [15–28]	24.01	5	<0.001	79.18	17.78	5	<0.001	71.87
Blood Biomarkers	0.60 [0.52–0.67]	0.82 [0.71–0.89]	3.2 [2.1–5.1]	0.50 [0.42–0.59]	7 [4–11]	3.06	3	0.38	1.86	10.63	3	0.01	71.77
Echocardiography	0.83 [0.72–0.91]	0.93 [0.89–0.96]	12.4 [6.8–22.9]	0.18 [0.10–0.32]	70 [23–213]	49.89	5	<0.001	89.98	114.39	5	<0.001	95.63
miRNA	0.87 [0.81–0.92]	0.66 [0.52–0.77]	2.6 [1.8–3.8]	0.19 [0.12–0.30]	13 [6–28]	3.7	4	0.45	0	0.22	4	0.99	0
Others (chest x-ray, clinical code, CT scan, and MRI)	0.79 [0.62–0.89]	0.85 [0.62–0.95]	5.3 [1.9–14.4]	0.25 [0.14–0.44]	21 [7–66]	142.16	5	<0.001	96.48	649.83	5	<0.001	99.23

**Figure 3:**
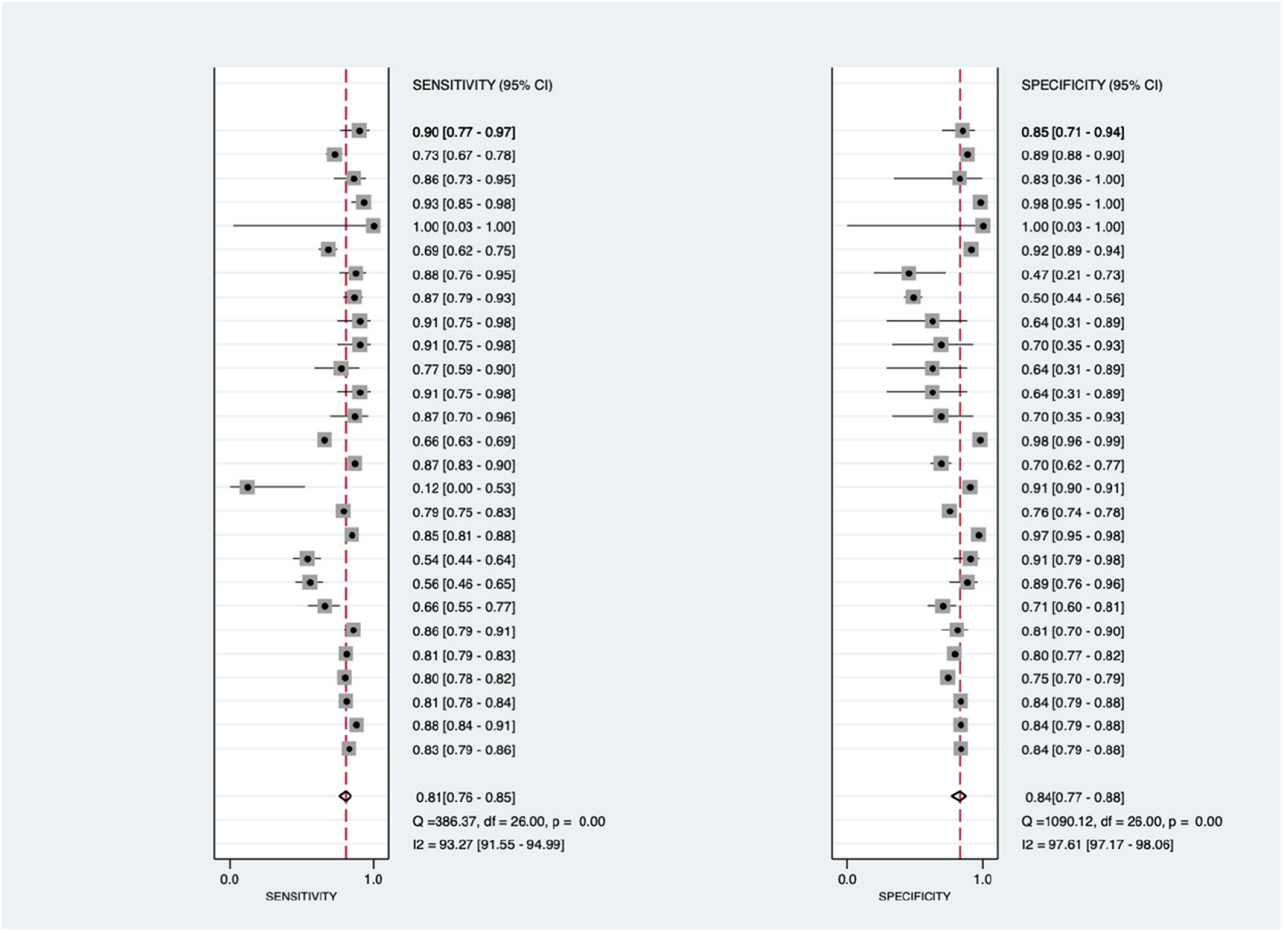
Forest plot showing overall sensitivity (left forest plot) and specificity (right forest plot) with corresponding heterogeneity statistics. The gray square and solid lines represent the odds ratio with 95% confidence intervals. The rhombus indicates the pooled estimate with 95% confidence intervals.

**Figure 4:**
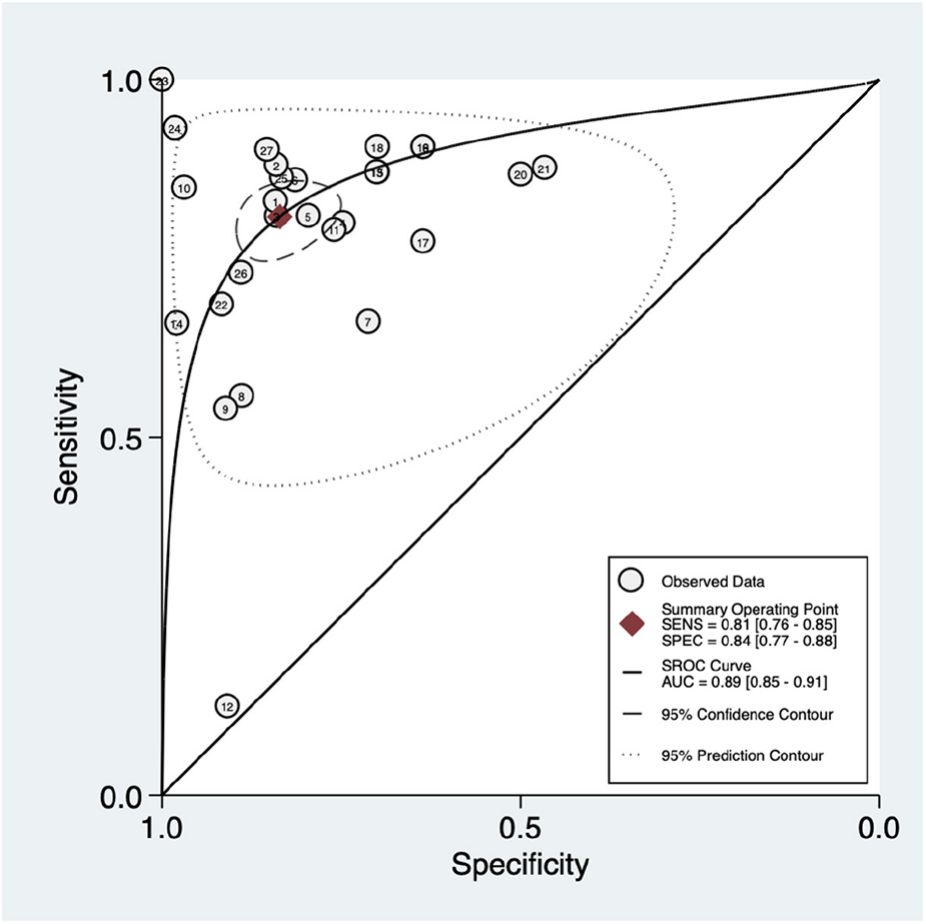
Summary ROC curve with confidence and prediction regions around mean operating sensitivity and specificity point.

As shown in Figure 3, there was significantly high heterogeneity in the pooled sensitivity [I^2 ^= 93.27%, *p* < 0.001] and specificity [I^2 ^= 97.61%, *p* < 0.001] values. Therefore, the random-effects model was used to analyze diagnostic parameters. The forest diagram shows the value of machine learning in the diagnosis of pulmonary hypertension; the pooled sensitivity was significant with a value of 0.81 [95% CI = 0.76–0.85, *p* < 0.001] and specificity was also significant with the value of 0.84 [95% CI = 0.77–0.88, *p* < 0.001]. In addition, Figure 4 shows a summary receiver operator characteristic (SROC) curve with an AUC of 0.89 [95% CI = 0.85–0.91].

**Figure 5:**
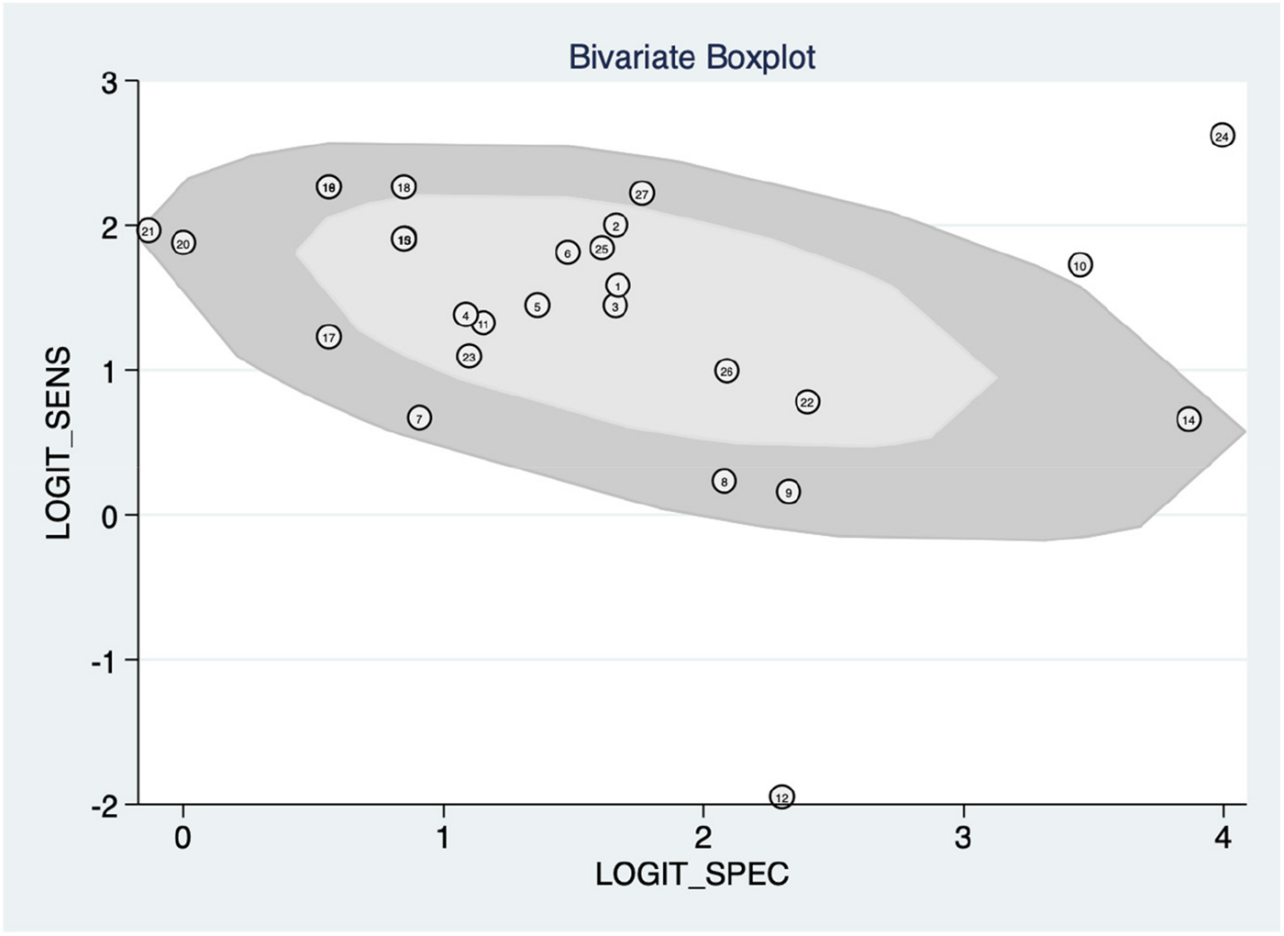
Bivariate boxplot with most studies clustering within the median distribution with three outliers suggesting indirectly a lower degree of heterogeneity.

Figure 5 shows the construction of a bivariate boxplot, which is a useful tool for detecting heterogeneity in each study. One study did not occur in the boxplot, including study 13 meanwhile three studies presented as outliers, including studies 10 (22), 12 (13), and 24 (12). Study 12 (23) involved patients with PAH and other subtypes of PH, study 10 involved the use of Chest x-ray, study 24 involved the use of ECG, meanwhile, study 12 involved the use of clinical code. This implies that the type of diagnostic method conducted prior to the machine learning algorithm could be the main cause of heterogeneity.

This study also employed Deek's funnel plot asymmetry test to assess the potential of publication bias between included studies. A value of *P* = 0.67 indicates a symmetric funnel shape, suggesting the absence of publication bias within the dataset under examination. This finding implies that the distribution of studies across the range of effect sizes is balanced and unbiased, contributing to the reliability and robustness of the analysis.

### Subgroup diagnostic performance analysis

Then, subgroup analysis was performed based on the diagnostic type of machine learning. Results are shown in Table 1. Concerning the diagnostic type, miRNA studies exhibited the highest sensitivity (0.87; 95% CI = 0.81–0.92; *p* = 0.45), however, the result was not statistically significant. Echocardiography followed with the second-highest sensitivity and yielded statistically significant results (0.83; 95% CI = 0.72–0.91; *p* < 0.001). The echocardiography also showed the highest specificity (0.93; 95% CI = 0.89–0.96; *p* = <0.001). The highest positive likelihood ratio (PLR) with a value of 12.4 (95% CI = 6.8–22.9) is also shown by echocardiography and the highest negative likelihood ratio (NLR) with a value of 0.50 (95% CI = 0.42–0.59) was shown by blood biomarkers. Furthermore, echocardiography also exhibited the highest diagnostic odds ratio (DOR) with values of 70 (95% CI = 23–213).

**Figure 6:**
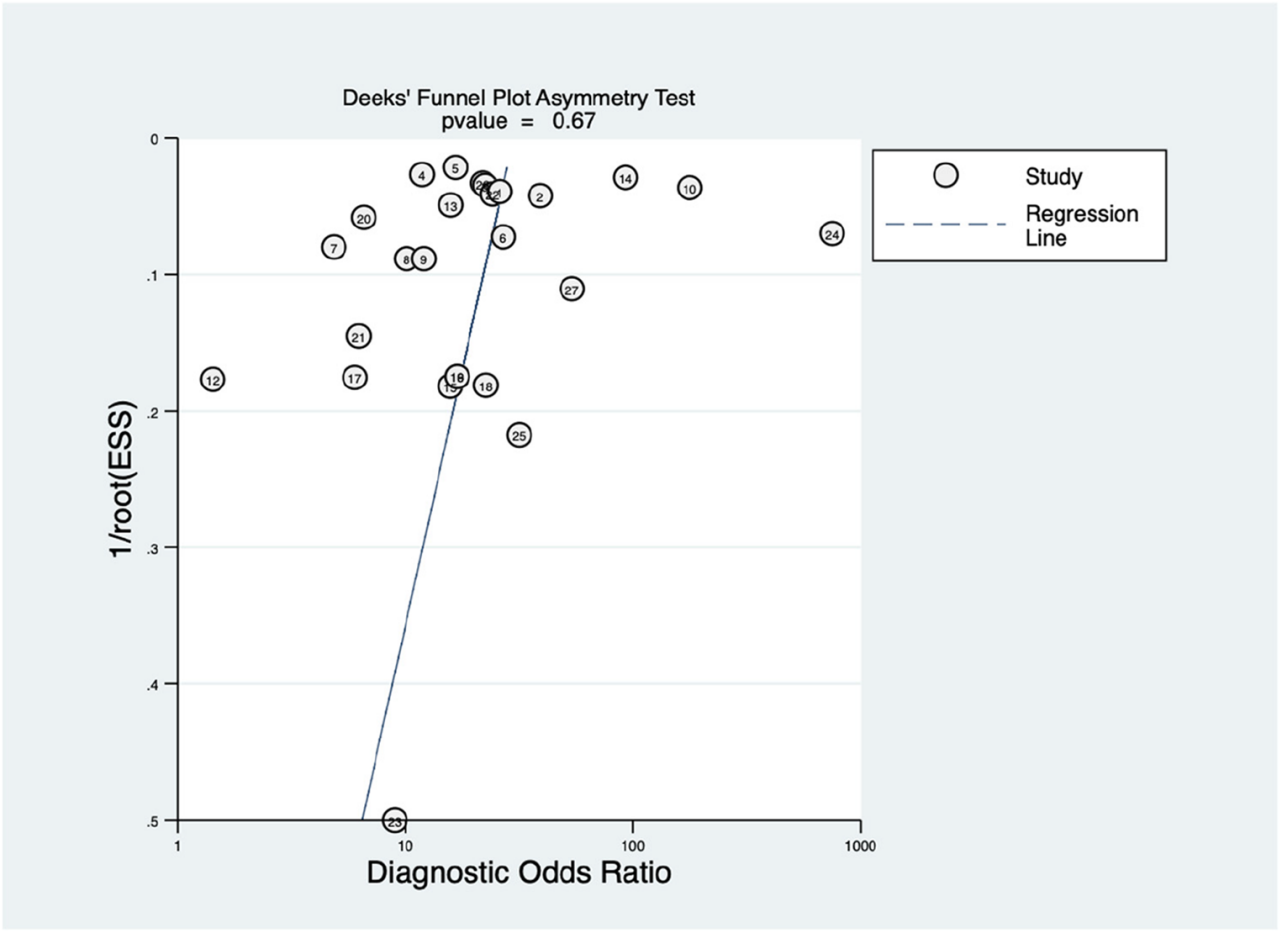
Funnel plot with superimposed regression line. The vertical axis displays the inverse of the square root of the effective sample size [1/root(ESS)]. The horizontal axis displays the diagnostic odds ratio (DOR). This Deek's funnel plot asymmetry test is a useful tool for assessing the potential publication bias in studies.

Figure 6 shows the diagnostic subgroup analysis of ECG as machine learning's diagnostic method. There was significantly high heterogeneity in the pooled sensitivity [*I*^2 ^= 79.18%, *p* < 0.001] and moderate heterogeneity in the pooled specificity [*I*^2 ^= 71.87%, *p* < 0.001] values. Therefore, the random-effects model was used to analyze diagnostic parameters. The forest diagram shows the value of machine learning in the diagnosis of pulmonary hypertension; the pooled sensitivity was significant with the values of 0.82 [95% CI = 0.80–0.84, *p* < 0.001] and specificity was also significant with the values of 0.82 [95% CI = 0.78–0.84, *p* < 0.001].

**Figure 7:**
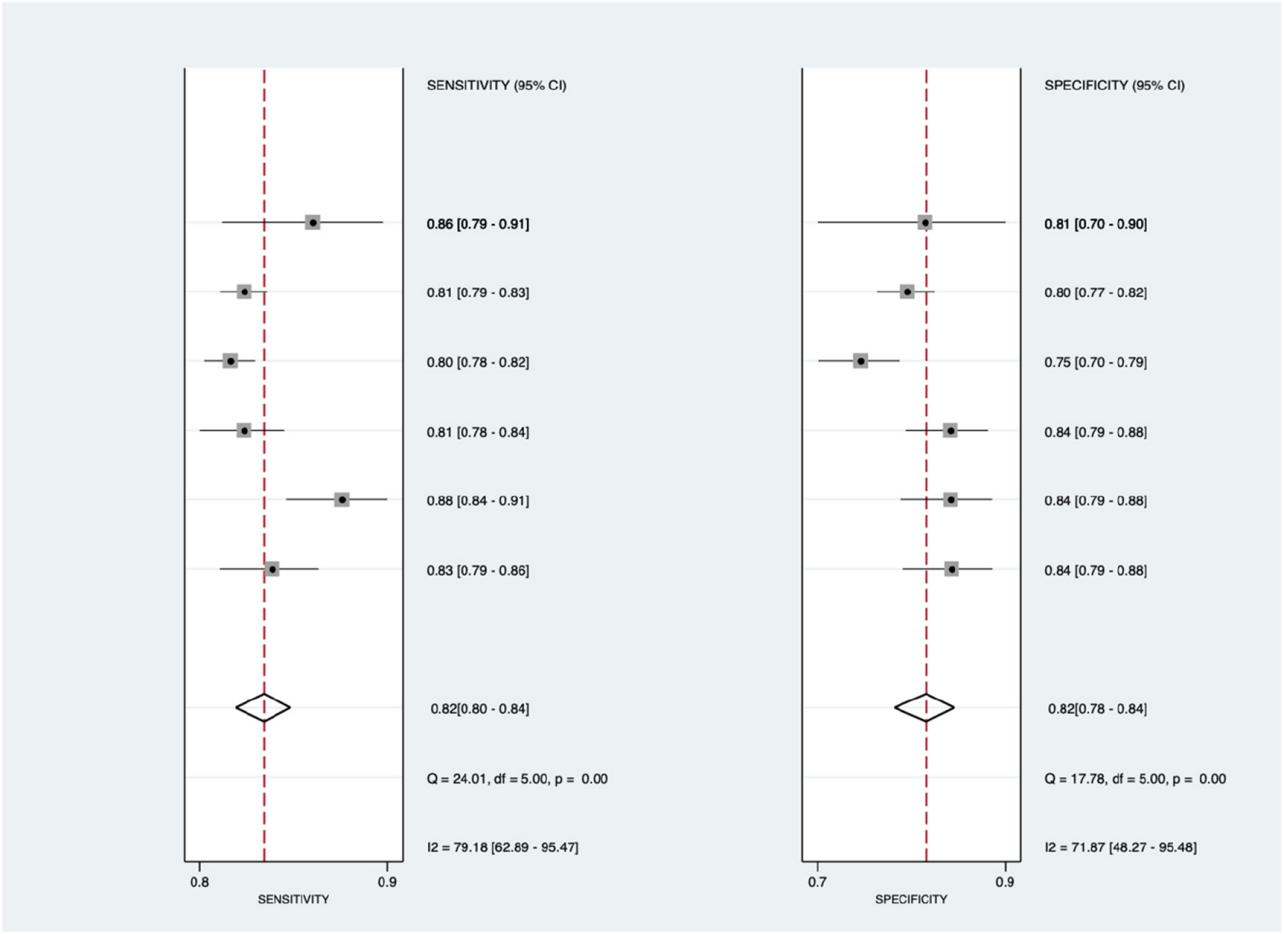
Forest plot showing echocardiography subgroup mean sensitivity (left forest plot) and specificity (right forest plot) with corresponding heterogeneity statistics. The gray square and solid lines represent the odds ratio with 95% confidence intervals. The rhombus indicates the pooled estimate with 95% confidence intervals.

Figure 7 shows the diagnostic subgroup analysis of echocardiography as machine learning's diagnostic method. There was significantly high heterogeneity in both the pooled sensitivity [*I*^2 ^= 89.98%, *p* < 0.001] and specificity [*I*^2 ^= 95.63%, *p* < 0.001]. Therefore, the random-effects model was used to analyze diagnostic parameters. The forest diagram shows the value of machine learning in the diagnosis of pulmonary hypertension; the pooled sensitivity was significant with values of 0.83 [95% CI = 0.72–0.91, *p* < 0.001] and specificity was also significant with values of 0.93 [95% CI = 0.89–0.96, *p* < 0.001].

**Figure 8:**
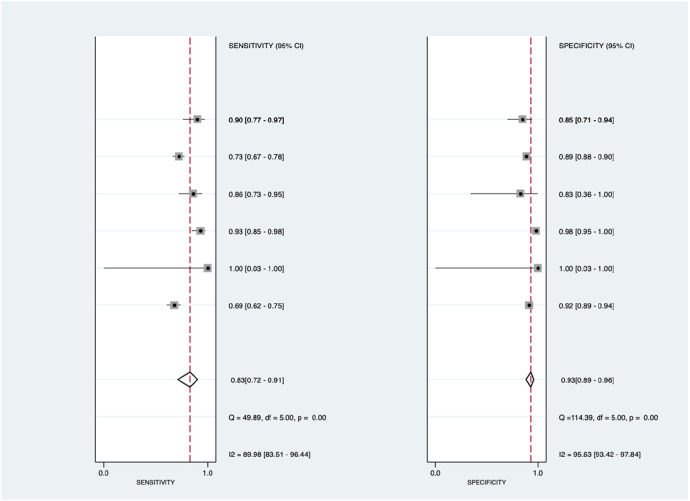
Forest plot showing blood biomarkers subgroup mean sensitivity (left forest plot) and specificity (right forest plot) with corresponding heterogeneity statistics. The gray square and solid lines represent the odds ratio with 95% confidence intervals. The rhombus indicates the pooled estimate with 95% confidence intervals.

Figure 8 shows the diagnostic subgroup analysis of blood biomarkers as machine learning's diagnostic method. There was no significant heterogeneity in the pooled sensitivity [*I*^2 ^= 1.86%] and significantly moderate heterogeneity in specificity [*I*^2 ^= 71.77%, *p* < 0.001] values. Therefore, the random-effects model was used to analyze diagnostic parameters. The forest diagram shows the value of machine learning in the diagnosis of pulmonary hypertension; the pooled sensitivity was insignificant with values of 0.60 [95% CI = 0.52–0.67, *p* = 0.38] and specificity was significant with values of 0.82 [95% CI = 0.71–0.89, *p* = 0.01].

**Figure 9:**
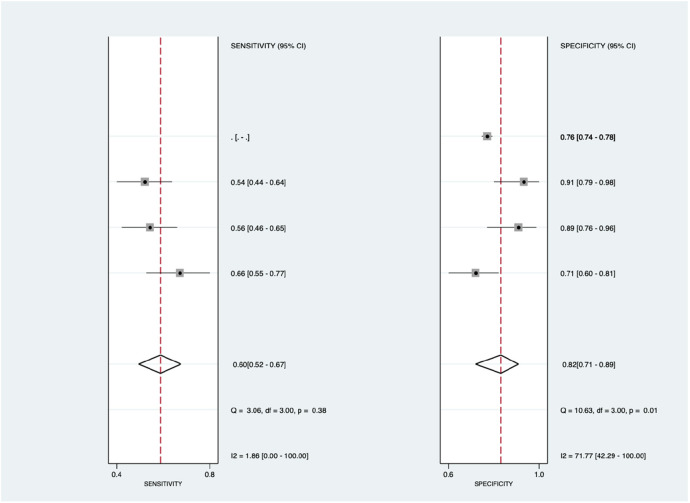
Forest plot showing microRNA subgroup mean sensitivity (left forest plot) and specificity (right forest plot) with corresponding heterogeneity statistics. The gray square and solid lines represent the odds ratio with 95% confidence intervals. The rhombus indicates the pooled estimate with 95% confidence intervals.

Figure 9 shows the diagnostic subgroup analysis of microRNA as machine learning's diagnostic method. There was nonsignificant heterogeneity in both the pooled sensitivity [*I*^2 ^= 0.00%] and specificity [*I*^2 ^= 0.00%] values. The forest diagram shows the value of machine learning in the diagnosis of pulmonary hypertension; the pooled sensitivity was insignificant with the values of 0.87 [95% CI = 0.81–0.92, *p* = 0.45] and specificity was also insignificant with the values of 0.66 [95% CI = 0.52–0.77, *p* = 0.99].

**Figure 10:**
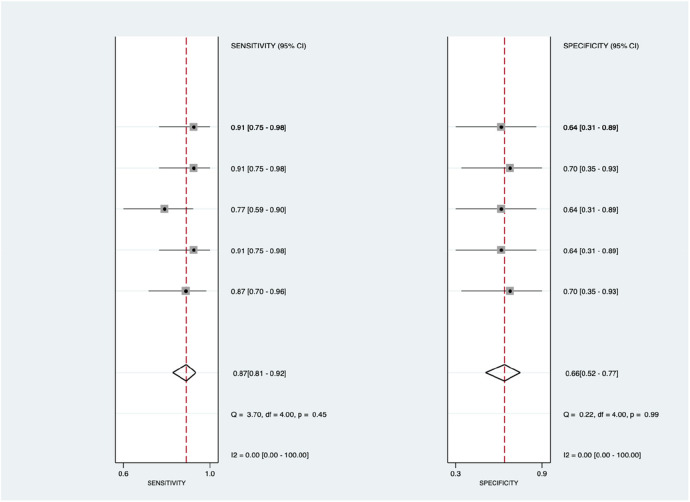
Forest plot showing other subgroups’ mean sensitivity (left forest plot) and specificity (right forest plot) with corresponding heterogeneity statistics. The gray square and solid lines represent the odds ratio with 95% confidence intervals. The rhombus indicates the pooled estimate with 95% confidence intervals.

Figure 10 shows the diagnostic subgroup analysis of others, including chest x-ray, CT scan, MRI, and clinical code, as machine learning's diagnostic method. There was a significantly high heterogeneity in both the pooled sensitivity [*I*^2 ^= 96.48%, *p* < 0.001] and specificity [*I*^2 ^= 99.23%, *p* < 0.001] values. Therefore, the random-effects model was used to analyze diagnostic parameters. The forest diagram shows the value of machine learning in the diagnosis of pulmonary hypertension; the pooled sensitivity was significant with values of 0.79 [95% CI = 0.62–0.89, *p* < 0.001] and specificity was also significant with values of 0.85 [95% CI = 0.62–0.95, *p* < 0.001].

### Diagnostic precision properties analysis

A single-group meta-analysis was utilized to analyze the diagnostic precision properties (Supplementary Table S2). Among the twenty studies assessing sensitivity, the overall estimated proportion was 77.98%, with a 95% CI ranging from 71.53% to 84.43% (Supplementary Figure S5). This suggests a high prevalence of sensitivity across the studies included, with the confidence interval indicating greater precision and statistical significance. High and significant overall heterogeneity was observed (*I*^2^ = 100%; *P* < 0.01).

For specificity, twenty studies were examined, revealing an overall estimated proportion of 79.93%, with a 95% CI between 73.66% and 85.01% (Supplementary Figure S6). Similar to sensitivity, there was a high prevalence of specificity, supported by a precise and statistically significant confidence interval. Overall heterogeneity was also high and significant (*I*^2^ = 100%; *P* < 0.01).

In the case of the AUC, fourteen studies contributed to an overall estimated proportion of 87.94%, with a 95% CI from 85.07% to 89.70% (Supplementary Figure S7). This indicates a high prevalence of AUC, with a precise and statistically significant confidence interval. Overall heterogeneity remained high and significant (*I*^2^ = 97%; *P* < 0.01).

Regarding the ROC, six studies were analyzed, resulting in an overall estimated proportion of 87.25%, with a 95% CI between 81.76% and 91.27% (Supplementary Figure S8). Again, a high prevalence of ROC was observed, supported by a precise and statistically significant confidence interval. Overall heterogeneity was high and significant (*I*^2^ = 92%; *P* < 0.01).

For positive predictive value (PPV), ten studies were considered, revealing an overall estimated proportion of 84.09%, with a 95% CI ranging from 57.18% to 95.44% (Supplementary Figure S9). A high prevalence of PPV was found, along with a precise and statistically significant confidence interval. Overall heterogeneity remained high and significant (*I*^2^ = 100%; *P* < 0.01).

Lastly, ten studies contributed to the analysis of negative predictive value (NPV), resulting in an overall estimated proportion of 93.78%, with a 95% CI from 81.17% to 98.14% (Supplementary Figure S10). Similar to the other metrics, a high prevalence of NPV was observed, supported by a precise and statistically significant confidence interval. Overall heterogeneity was once again high and significant (*I*^2^ = 100%; *P* < 0.01).

## Discussion

### Conventional method in diagnosing pulmonary arterial hypertension

Pulmonary hypertension (PH) encompasses a spectrum of conditions characterized by elevated blood pressure in the pulmonary arteries, with pulmonary arterial hypertension (PAH) being a distinct subgroup primarily affecting the small pulmonary arterioles. PAH presents significant challenges in diagnosis due to its multifaceted etiology and diverse clinical manifestations (24) To accurately diagnose PAH and differentiate it from other forms of PH, a comprehensive diagnostic approach combining clinical assessment, imaging modalities, and invasive procedures is necessary.

Clinical evaluation forms the cornerstone of PAH diagnosis, involving a detailed medical history, physical examination, and assessment of symptoms. Symptoms of PAH can vary widely, ranging from early indicators such as exertional dyspnea and fatigue to more advanced symptoms like exertional chest pain and syncope. Recognizing these symptoms alongside physical examination findings, such as an enlarged right ventricle or abnormal heart murmurs, provides crucial initial insights into the possibility of PAH (24, 25).

Diagnostic imaging plays a pivotal role in identifying structural and functional abnormalities associated with PAH. Electrocardiography (ECG or EKG) provides valuable information by detecting electrical abnormalities indicative of right ventricular hypertrophy and strain, common features of PAH. Transthoracic echocardiography (TTE) offers non-invasive assessment of pulmonary artery pressures and right heart function, aiding in the diagnosis and monitoring of PAH progression. Chest x-rays complement these assessments by visualizing cardiac and pulmonary structures, revealing characteristic findings such as central pulmonary arterial dilatation and signs of right heart enlargement (24).

While non-invasive tests provide valuable diagnostic information, invasive procedures such as right heart catheterization (RHC) remain the gold standard for confirming PAH diagnosis. RHC enables direct measurement of pressures in the heart and pulmonary arteries, including mean pulmonary arterial pressure (mPAP) and pulmonary vascular resistance (PVR). These hemodynamic parameters aid in distinguishing between pre-capillary and post-capillary PH and guide therapeutic decision-making. Post-capillary PH is defined by hemodynamic measurements of mPAP greater than 20 mmHg and pulmonary artery wedge pressure (PAWP) greater than 15 mmHg (25).

Laboratory tests, including hematological assessments, contribute additional diagnostic insights by identifying specific biomarkers associated with PAH, such as brain natriuretic peptide (BNP) or N-terminal pro-brain natriuretic peptide (NT-proBNP). Elevated levels of these biomarkers may indicate cardiac stress and provide prognostic information in PAH (26).

Despite advancements in diagnostic techniques, challenges persist in optimizing early detection and improving prognostication in PAH. Future directions in PAH diagnosis may involve the integration of novel biomarkers, advanced imaging technologies, and machine learning algorithms to enhance diagnostic accuracy and tailor treatment strategies to individual patient needs.

### General mechanism of machine learning

ML forms the core of artificial intelligence (AI), enabling computers to handle complex tasks using an array of advanced methods (25, 27). ML harnesses algorithms that elucidate relationships between variables to drive accurate predictions (28). The fundamental premise of machine learning is to enable computers to improve their performance on a specific task over time as they are exposed to more data through training (29).

The landscape of ML methods is diverse, but they broadly fall into two categories: supervised and unsupervised learning (30). Supervised learning involves training models on inputs linked with known outcomes. For example, in medical diagnosis, models can be trained on various patient characteristics to predict the onset of diseases. Supervised algorithms are meticulously developed using datasets containing multiple variables and relevant outcomes. However, the risk of overfitting, where the model overly tailors itself to the training data, necessitates careful validation through techniques like splitting datasets into training and testing segments. In each segment, there is a randomly chosen portion of features along with their corresponding outcomes. This enables the algorithm to link specific features or traits to outcomes, a process known as algorithm training. Following training, the algorithm is applied to the features in the testing dataset without their corresponding outcomes. The predictions generated by the algorithm are subsequently compared to the known outcomes of the testing dataset to evaluate model performance. This step is crucial for enhancing the algorithm's ability to effectively generalize to new data (28). On the other hand, unsupervised learning ventures into uncharted territory, seeking patterns and clusters within datasets without predefined outcomes. These techniques, while exploratory, offer invaluable insights into complex data structures (28, 31, 32).

In the domain of medical diagnosis, leveraging ML entails a complex sequence of procedural steps. It commences with data acquisition, encompassing diverse sources like clinical records, imaging, and patient histories. Subsequently, data undergoes processing, addressing issues such as missing values and noisy data. Identification of target variables and predictors follows suit, paving the way for model training. Once trained, these models serve as powerful diagnostic tools, aiding healthcare professionals in making informed decisions (5).

Essentially, the framework of machine learning surpasses mere algorithms; it embodies a comprehensive approach that spans data acquisition, processing, model training, and practical application. As ML continues to develop, its integration into various domains promises transformative outcomes, revolutionizing how complex tasks are undertaken and decisions are made.

### Current Use of machine learning in medical fields

In recent years, artificial intelligence (AI) and machine learning (ML) have emerged as potent tools across various domains, promising transformative solutions to complex challenges (33). Specifically, within medical field, ML-assisted diagnosis stands out as a potential game-changer, leveraging vast patient datasets to deliver precise and personalized diagnoses. Despite considerable research and commercial interest, diagnostic algorithms helped to increase the diagnostic accuracy of human doctors in scenarios involving multiple potential causes for a patient's symptoms.

Medical diagnosis is inherently intricate, with numerous factors such as overlapping structures, distractions, fatigue, and limitations of the human visual system contributing to potential misdiagnosis. ML methods have been increasingly adopted to help clinicians in overcoming these challenges, facilitating informed and accurate decision-making in disease diagnosis (5). By employing intelligent data analysis tools, ML helps unveil intricate relationships within datasets, providing valuable second opinions to clinicians and potentially improving patient outcomes while reducing treatment costs.

ML techniques hold promise in two key areas within medical practice: diagnosis and outcome prediction. These methods have demonstrated success in tasks such as classifying skin cancer from images and predicting the progression from pre-diabetes to type 2 diabetes using electronic health record data (28). Moreover, ML is finding increasing application in cardiovascular disease diagnostics, spanning modalities such as echocardiography, electrocardiography, and non-invasive imaging (34, 35).

In the realm of medical imaging, ML algorithms play a pivotal role in enhancing the detection and diagnosis of conditions from x-rays, CT scans, and MRIs, aiding in the identification of tumors, fractures, and anomalies (34, 36). Additionally, ML is instrumental in genomic data analysis, facilitating the detection of disease-related patterns and mutations, thereby offering insights into individual responses to treatments (Wu and Zhao, 2019).

Furthermore, ML contributes to drug discovery by elucidating disease molecular mechanisms and predicting potential medication candidates, thereby enhancing efficiency and quality in lead synthesis pathways (31, 37). ML-driven decision support systems analyze patient data extracted from electronic health records (EHRs), aiding healthcare providers in identifying issues, suggesting remedies, and assessing illness probabilities (5).

Beyond diagnosis and treatment, ML models analyze extensive datasets to identify trends and patterns in disease occurrence, informing preventive measures in public health (dos Santos et al., 2019). Additionally, ML-based predictive models analyze behavioral patterns and social media data to predict mental health conditions, with ML-powered chatbots and virtual therapists offering support and counseling (38).

However, integrating ML into healthcare necessitates careful consideration of data privacy, model interpretability, and ethical concerns despite its potential to revolutionize healthcare delivery (39). As research and technological advancements continue, the field of ML in healthcare is expected for further advancement and innovation.

### Precision of machine learning in diagnosing pulmonary arterial hypertension

#### Overall diagnostic performance analysis

This study assesses overall diagnostic performance machine learning in diagnosing PAH using several methods (data source) of PAH diagnostic, which are ECG, Echo, blood biomarkers, miRNA, and other (unclassified) diagnostic methods (x-ray, clinical code, MRI, and CT scan). The results of this study found that although significant heterogeneity is found, the diagnostic approach using machine learning is significant and specific with an excellent value of SROC (0.8–0.9 is excellent, while more than 0.9 is outstanding). However, the heterogeneity is found due to the variety of machine learning methods employed in the analysis.

By analyzing various data types such as medical images, patient records, and biomarkers, machine learning algorithms can identify patterns and correlations that may not be apparent to human observers. Therefore, the usage of machine learning is remarkable in increasing sensitivity and specificity compared to conventional methods of diagnosis. However, it's essential to recognize that the performance of machine learning models depends on the quality and quantity of the data used for training. Due to the heterogeneity of the data source, subgroup analysis is done to compare the quantity and quality of the data itself.

In a study by Bauer et al. in 2020, it is stated that machine learning demonstrated the potential of machine learning algorithms in predicting outcomes in PH patients based on variables such as demographic information, comorbidities, and hemodynamic parameters (21). Another study by Swift et al. (2021) shows the superiority of automatic detection and segmentation of the ventricles using machine learning which makes data derived from these approaches may reduce the need for manual adjustments that are currently labour intensive, especially for the right ventricle and therefore, increasing the accuracy of the diagnostic method (40).

Machine learning algorithms excel at identifying patterns within extensive datasets. Diagnosing pulmonary arterial hypertension (PAH) involves assessing multiple clinical parameters like echocardiography results, pulmonary function tests, blood biomarkers, and patient history. ML models can thoroughly analyze these diverse data points, detecting nuanced patterns that traditional diagnostic approaches might miss (6, 41). ML algorithms are proficient at integrating data from various sources, including imaging studies, clinical assessments, and laboratory tests. They excel at managing multimodal data, enabling a comprehensive analysis that encompasses all pertinent information for diagnosing PAH (7, 42).

#### Subgroup diagnostic performance analysis

Echocardiography showed outstanding results on sensitivity, specificity, PLR, and DOR with statistically significant results compared to other diagnostic methods. With 83% sensitivity and 93% specificity, echocardiography showed great precision in diagnosing PAH. This finding is aligned with previous studies, which stated that echocardiography is superior in diagnosing PAH (11, 43, 44).

Blood biomarkers yielded the most favorable result for NLR (0.50), indicating their capability to accurately identify patients as negative for PH. However, blood biomarkers displayed moderate sensitivity at 60%. A high negative likelihood ratio (LR-) combined with low sensitivity suggests that while the test is adept at correctly identifying individuals without the condition (true negatives), it may be less effective at detecting those who actually have the condition (false negatives). Essentially, although the test excels in ruling out the presence of the condition in healthy individuals, it may frequently miss detecting the condition in those who are afflicted. This situation could be attributed to various factors such as limitations of the test, inherent variability in the condition being examined, or the influence of confounding variables on the test outcomes (45, 46).

When 80% is set as a cutoff value of excellent sensitivity and specificity, ECG is still considered a precise method to diagnose PAH with 82% and 82%. This finding is aligned with previous studies, which stated that ECG in combination with machine learning is able to increase the diagnostic sensitivity and specificity, even using fewer than 12 leads ECG. Since electrocardiograms (ECGs) are widely available in clinical settings, it's also feasible that this algorithm could be utilized in primary care or resource-limited environments (47). According to a study by Kwon et al. (2020), the dependable performance of an AI algorithm based on a single-lead ECG suggests the potential for screening pulmonary arterial hypertension (PAH) using both standard 12-lead ECGs and simpler wearable or monitoring devices. The study also highlights the significant diagnostic accuracy achieved by combining ECG data with machine learning techniques (15). This could potentially expedite echocardiographic assessments, diagnoses, and referrals to specialists.

Although miRNA deployed excellent sensitivity (87%) and moderate specificity (66%), both results are considered to be statistically insignificant. This might develop from the minimal number of studies and variations in machine learning software. The “others” category demonstrated impressive sensitivity (79%) and specificity (85%). However, it is important to highlight that this category includes chest x-ray, clinical code, CT scan, and MRI, making it impossible to analyze these diagnostic methods separately. Future research should aim to individually assess these diagnostic methods to thoroughly evaluate their diagnostic efficacy.

### Non-Diagnostic (single-arm) meta-analysis of diagnostic parameters analysis

This study assesses diagnostic parameters of the machine learning performance using non-diagnostic (single-arm) meta-analysis which is able to display each of the study's proportions in assessing the significance of a certain parameter. Each assessment of a specific parameter's efficacy is supplemented with a subgroup examination aimed at elaborating the diagnostic technique utilized.

**Figure 11:**
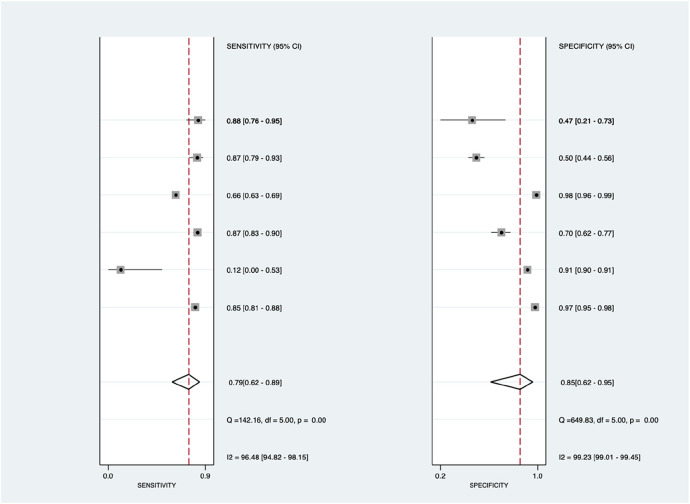
Forest plot showing ECG subgroup mean sensitivity (left forest plot) and specificity (right forest plot) with corresponding heterogeneity statistics. The gray square and solid lines represent the odds ratio with 95% confidence intervals. The rhombus indicates the pooled estimate with 95% confidence intervals.

In sensitivity parameter, the study identified significant findings with variations from high to low heterogeneity across different categories, namely clinical codes, blood biomarkers, ECG, and miRNA while the heterogeneity of chest x-ray and CT scan cannot be assessed due to the minimal studies conducted regarding this mode of diagnosis. miRNA displayed the lowest heterogeneity (11%) due to the same gene sequence used with the main difference in the type of software utilized (48). High heterogeneity found in other studies might depict the variations of patients' baseline characteristics, different approaches in conducting the main diagnostic method, or the variety of machine learning software used. Similar results were found in Figure 11 (specificity), Figure 6 (AUC), Figure 8 (PPV), and Figure 9 (NPV). The random forest method was particularly effective in pinpointing patients with pulmonary arterial hypertension (PAH) with high sensitivity, although XGBoost also yielded a similarly strong Area Under the Curve (AUC). One specific microRNA, MiR-187, stood out in this study and was notably upregulated in samples from endoarterial biopsies in a porcine model. This suggests that MiR-187-5p and MiR-636, identified as potential biomarkers, could be linked to the progression of PAH. This validation underscores the relevance of our machine learning approach in identifying microRNA biomarkers, indicating their potential utility as personalized prognostic indicators (48).

However for the study depicted in Figure 7 (ROC), significant findings were identified with lowest heterogeneity in blood biomarkers method. The diagnostic characteristics of biomarkers are deemed vital for enhancing accuracy, particularly in processes like pulmonary vascular remodeling involving proteins such as RAGE and MMP-2, angiogenesis, and cellular growth involving collagen IV, endostatin, IGFBP-2, and neuropilin-1, as well as cardiac dysfunction marked by NT-proBNP and IGFBP-7. RAGE, among the highest-ranked proteins, holds significance in accumulating extracellular matrix proteins, particularly influencing vascular remodeling (49–51). However, the usage of other methods are also significant considering its proportional efficacy and significance in diagnosing PAH, especially due to its personalized approach in diagnosing (3, 44).

### Benefits and implications of implementing machine learning-based diagnostic method for diagnosing pulmonary arterial hypertension

Machine learning plays a crucial role in early diagnostic approaches for patients with pulmonary arterial hypertension (PAH). By analyzing extensive datasets encompassing patient medical history, symptoms, and diagnostic tests, ML can discern patterns suggestive of PAH (11). This early detection facilitates timely intervention, ultimately improving patient outcomes (40). Moreover, machine learning enhances the accuracy of diagnosing pulmonary hypertension by scrutinizing intricate data from diverse sources. Through this, it tailors treatment plans to individual patients, constituting personalized medicine. Consequently, treatment efficacy is potentially heightened, and adverse effects are mitigated. Furthermore, machine learning serves as a risk stratification tool by evaluating various factors to gauge the likelihood of disease progression and complications in pulmonary hypertension patients (2, 40). This empowers healthcare providers to prioritize high-risk individuals for vigilant monitoring and proactive intervention (21, 40).

In the realm of pulmonary hypertension, machine learning algorithms exhibit the capability to forecast the condition with remarkable precision by leveraging a wider array of echocardiographic data, eliminating the need for estimated right atrial pressure as a reliance factor (6). ML algorithms can effectively predict pulmonary hypertension in patients with invasively determined pulmonary artery pressure, potentially improving decision-making in PAH treatment (6). Additionally, deep learning algorithms demonstrate the capacity to precisely detect anomalies suggesting pulmonary hypertension in chest radiographs, exhibiting both high accuracy and broad applicability. This presents a hopeful, non-invasive, and easily accessible method for screening patients (22). Machine learning algorithms trained on large datasets can estimate prognosis and potentially guide therapy in adult congenital heart disease (ACHD (11, 52). In the field of cardiology, machine learning techniques can also enhance efficiency by optimizing performance and extracting valuable data from both contrast-enhanced cardiac CT angiography (CCTA) and non-contrast enhanced cardiac CT scans. This improvement in diagnostic accuracy also holds significant implications for prognosis (34).

### Study strengths and limitations

This diagnostic meta-analysis provides a comprehensive investigation into the diagnosis of PAH by employing the combination of multiple non-invasive diagnostic techniques augmented with ML algorithms, based upon the latest studies. This study serves as the first diagnostic meta-analysis to evaluate this progression, based on the availability of studies in scholarly databases and the PROSPERO registry. Additionally, it delves into the individual diagnostic performance of each method, aiding in the identification of superior diagnostic approaches. Nonetheless, the study is not without limitations; notably, there is an uneven distribution of studies across different diagnostic methods, with a predominant focus on ECG and echocardiography. Moreover, a comparison of diagnostic performance with RHC considered the gold standard, was precluded due to the unavailability of studies directly comparing ML-based diagnostic methods with RHC in diagnosing PAH. While echocardiography has shown some encouraging results, further study comparing these results to RHC outcomes and to readings from patients with a variety of heart diseases is necessary. The diagnostic use of echocardiography for PAH will be better understood with the aid of this more comprehensive comparison of its sensitivity and specificity. Another limitation is, there is no study including any Doppler imaging modalities as its imaging modality due to lack of study. Therefore, we only included secondary signs of it and recommend further researchers to conduct primary research using machine learning and Doppler imaging modality.

## Conclusion

The integration of echocardiography and ECG with ML techniques for the diagnosis of PAH shows a promising avenue in non-invasive diagnostic strategies, potentially serving as a viable alternative to RHC as the gold standard. This innovative approach demonstrates considerable potential by yielding outstanding diagnostic outcomes, thereby fostering the development of more accessible and less invasive diagnostic modalities. While ECG and echocardiography are advancing, they do not replace RHC’s direct pressure measurements, despite its limitations. Nonetheless, further primary research is imperative, particularly in comparing combination of ML-based echocardiography and ECG with RHC in diagnosing PAH.

## Data Availability

The original contributions presented in the study are included in the article/Supplementary Material, further inquiries can be directed to the corresponding authors.
