# Supplementary material for: Assessing the precision of machine learning for diagnosing pulmonary arterial hypertension: a systematic review and meta-analysis of diagnostic accuracy studies
Source: Front Cardiovasc Med. 2024 Aug 27;11:1422327. doi: 10.3389/fcvm.2024.1422327 (PMC11385608; doi:10.3389/fcvm.2024.1422327)
Supplement: Supplementary file 1 [file Datasheet1.pdf]

SUPPLEMENTARY MATERIAL

Supplementary Table 1. Demography and clinical characteristics of included studies

No.	Author, Year	Study Design	Country	Center	Duration (months)	Age Mean ± SD/Median (IQR)		Sample Size (M/F)		Type of Disease	Type of Intervention	Diagnostic Method	Control
						Intervention	Control	Intervention	Control				
1	Aras et al., 2023[47]	Cohort Retrospective	USA	Single	84	Precapillary PH (by RHC only) = 58.4 ± 14.5<br>Group 1 PH - PAH = 55.0 ± 14.6<br>Group 3 PH - PH Owing to Lung Disease and/or Hypoxia = 62.5 ± 12.4		Precapillary PH (by RHC only) = 296/405<br>Group 1 PH - PAH = 237/335<br>Group 3 PH - PH Owing to Lung Disease and/or Hypoxia = 562/396		Precapillary PH, Group 1 PH, Group 3 PH	Keras version 2.2.4 and Python 3.6	ECG	Non-PH
2	Bauer et al., 2021[21]	Cohort Retrospective	Switzerland	Single	84	61.5±9.5	55.7±11.5	77	80	Group 1 PAH	Myriad DiscoveryMAP version 3.3, RF, and XGBoost	ECHO, ECG, RHC, Pulmonary Function Tests, NT-proBNP	Non-PH
3	Bordag et al., 2023[3]	Cohort Prospective	Austria	Single	72	66.5±3.0	59.5±4.7	74	30	Group 1-4 PH	Python 3.9, package Sklearn and XGBoost	Blood Samples	Lung Disease Controls withouth PH
4	Diller et al., 2022[11]	Cohort Retrospective	UK	Multi	156	59 [46–69]	40 [27–52]	450	308	PAH	U-Net architecture implemented in R/Keras	ECHO	RV Dilatation without PAH

5	Errington et al., 2021[48]	Cohort Retrospective	UK	Multi	72	56.5 (14.3)	54.1 (14.5)	18/24	11/18	Idiopathic PAH + Systemic Sclerosis(SScs)-PAH	Boruta (an embedded random forest method), LASSO, regression partition trees, and XGBoost (an extreme gradient boosting method)	miRNAs	Healthy Controls + SScs without PAH
6	Granot et al., 2021[4]	Cohort Retrospective	Israel	Single	47	77.78 (65.66–84.57)	64.71 (45.88–77.53)	100/183	93/169	PAH	Pulmonary Arterial Analysis, Extended Brilliance Workplace Portal v. 7	CT pulmonary angiography	Non-PAH
7	Hyde et al., 2023[44]	Cohort Retrospective	USA	Multi	47	69	71	849/490	2519/1703	PH	SHapley Additive exPlanations (SHAP)	Echocardiography	Non-PH
8	Imai et al., 2023[12]	Cohort Retrospective	Japan	Single	204	51.9 ± 16.1	67.3 ± 10.9	(34/111)	(131/129)	PAH	ResNet50 model pre-trained on ImageNet-1k	Chest Radiographs	Non-PAH
9	Kanwar et al., 2020[7]	Cohort Prospective	USA	Multi	12	53.6		506/2023		PAH	Tree Augmented Naïve Bayes model (PHORA)	NT-pro-BNP + 6MWD	NA
10	Kiely et al., 2019[13]	Cohort Retrospective	UK	Multi	204	60 ± 23	71 ± 23	(269/440)	(1,378,105/1,434,353)	Idiopathic PAH	Sheffield Pulmonary Hypertension IndeX (SPHInX)	Clinical Code	Non idiopathic PAH

11	Kogan et al., 2023[19]	Cohort Retrospective	USA	Multi	144	51.55 ± 15.64	61.50 ± 17.08	(392/562)	(5,259,222/6,010,642)	PH	XGBoost	Clinical Code (SHAP Value)	Healthy
12	Kusunose et al., 2022[14]	Cohort Prospective	Japan	Single	48	60 ± 14	57 ± 13	(9/43)	(8/81)	Exercise-induced PH	Chainer (ver. 7.2.0) deep learning framework	Echocardiography	Non exercise-induced PH
13	Kwon et al., 2020[15]	Cohort Prospective	Korea	Multi	36	Hospital A: 67.51 (14.09) Hospital B: 69.17 (15.12)	Hospital A: 59.17 (15.31) Hospital B: 57.46 (15.04)	Hospital A: (1,495/1,842) Hospital B: (334/423)	Hospital A: (11,992/12,045) Hospital B: (5,101/5,007)	PH	TensorFlow	ECG	Non-PH
14	Leha et al., 2019[6]	Cohort Retrospective	UK & Germany	Multi	60	68 ± 14	54 ± 19	(32/36)	(8/14)	PH	Support vector machine (SVM), lasso penalized logistic regression, boosted classification tree models using Quinlan's C5.0 algorithm, random forest of classification trees, and random forest of regression trees	Echocardiography	Non-PH

15	Liao et al., 2023[32]	Cohort Prospective	China	Multi	26	42 ± 15	40 ± 15	(21/56)	(17/56)	PH	CatBoost	Echocardiography	Non-PH
16	Liu et al., 2022[53]	Cohort Retrospective	Taiwan	Single	84	73.0 ±1 4.9; (Group 1 60.0 ±18.6);	59.0 ± 18.4	2,824	38,273	PH (type 1,2,3,4,5)	AI Model (Unspecified)	ECG and echocardiography	Non-PH (non elevated PAP)
17	Lungu et al., 2016[43]	Cohort Prospective	Sheffield, UK	Single		64 ± 16 (23)	56 ± 16 (29)	57	15	PH	Random forest classification using MATHLAB	ECHO	NA
18	Ong et al., 2020[23]	Cohort Retrospective	USA	Multi	84	74.3	73.1	389	161	PH subtypes (PAH and other PH subtypes)	Penalized lasso regression, random forest, and gradient boosting machine.	Electronic Medical Records (EMR)	Non-PH
19	Schuler et al., 2022[20]	Cohort Retrospective	USA	Single	NA	52.0 (13.7) 52.0 (21.4) 50.0 (15.0) 50.1 (14.4) 50.1 (17.1), RF Algorithm in SD RF Algorithm SD Non-Cases French Registry REVEAL UK-Ireland Registry,		653	1041	PAH	Elastic net, random forests (RF), extreme gradient boosting (XGBoost).	Electronic Medical Records (EMR)	Non-PAH
20	Seidler et al., 2019[16]	Cohort Prospective	Germany	Single	NA	NA	NA	90		PH	Random forest of classification trees, random forest of	Pulmonary artery pressure (PAP) with corresponding	NA

											regression trees, lasso penalized logistic regression, boosted classification trees, support vector machines	echocardiographic estimations of PAP obtained within 24 hours	
21	Sun et al., 2023[54]	Cohort Prospective	China	Multi	18	>18		2364	14698	PAH	Chamber Attention Network (CAN)	Echocardiography	Non-PH
22	Suvon et al., 2022[17]	Cohort Retrospective	Sheffield, UK	Single	144	NA	NA	233/233	233/2330	One Year Mortality in PAH patients	Bidirectional Encoder Representations from Transformers (BERT).	Echocardiography	Negative samples
23	Zeng et al., 2021[18]	Cohort	GSE117261 and GSE48149 datasets	NCBI Gene Expression Omnibus	NA	NA	NA	NA	NA	Idiopathic PAH	R software and the “clusterProfiler” package (19) were used to conduct	Biomarker	NA

				(GEO) database							GO analysis and KEGG analysis.		
24	Zhang et al., 2023[1]	Cohort Retrospective	China	Single	50	47.75 ± 14.87		55/84		PH (PAP Parameters)	XGBoost	CTPA	NA
25	Zou et al., 2020[22]	Cohort Retrospective	China	Multi	77	67.5 ± 14.8	49.4 ± 16.5	405	357	PH (PASP Value)	Resnet50, Xception, and Inception V3	Chest Radiographs	ImageNet (natural images)
26	Ge et al., 2023[55]	Cohort Prospective	China	Single	NA	NA		161	161	CHD-PAH	XGBoost	Echocardiography	CHD Non PAH

Supplementary Table 2. Additional Explanation for Overall Diagnostic Performance Analysis

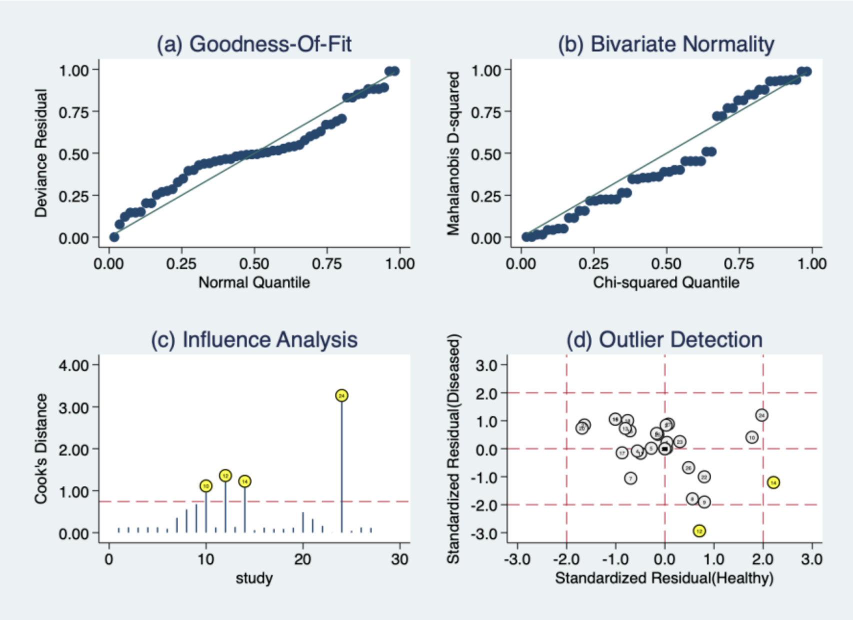

Supplementary Figure 1. Graphical depiction of residual-based (A) goodness-of-fit which is a statistical hypothesis test to see how well sample data fit a distribution from a population and shows normal distribution which is also supported by (b) bivariate normality. This figure also shows (c) influence analysis which shows the outlyingness, leverage, and influence of each case. The plot shows the residual on the vertical axis, leverage on the horizontal axis, and the point size is the square root of Cook's D statistic, a measure of the influence of the point. In addition, an (d) outlier detection analyses also show the outlier of each study by using the proximity of data points within two or three dimensions to identify outliers.

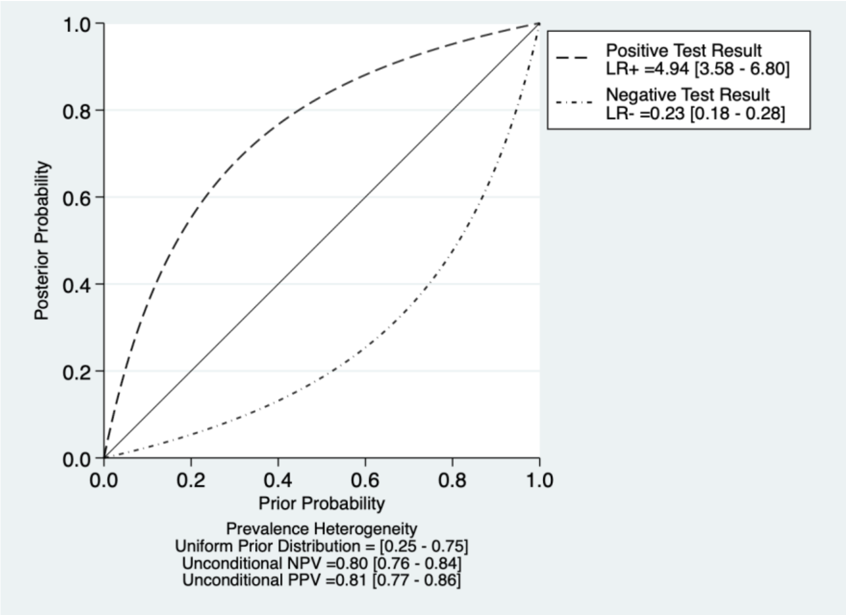

Supplementary Figure 2. Probability Modifying Plot with PLR of 4.94 [3.58 - 6.80] and NLR of 0.23 [0.18 - 0.28]. The points located along the probability plot line represent "normal," common, and random variations. The points at the upper or lower extreme of the line, or which are distant from this line, represent suspected values or outliers.

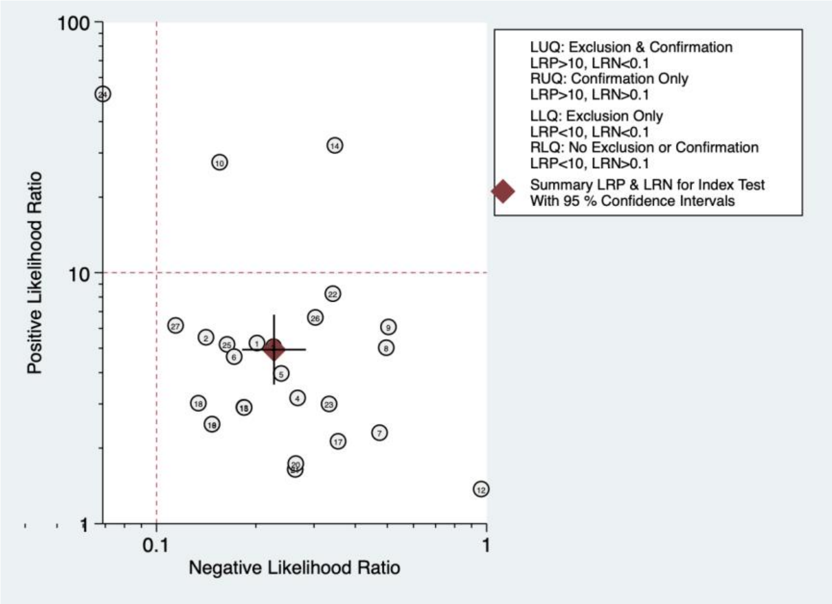

Supplementary Figure 3. Likelihood Ratio Scattergram

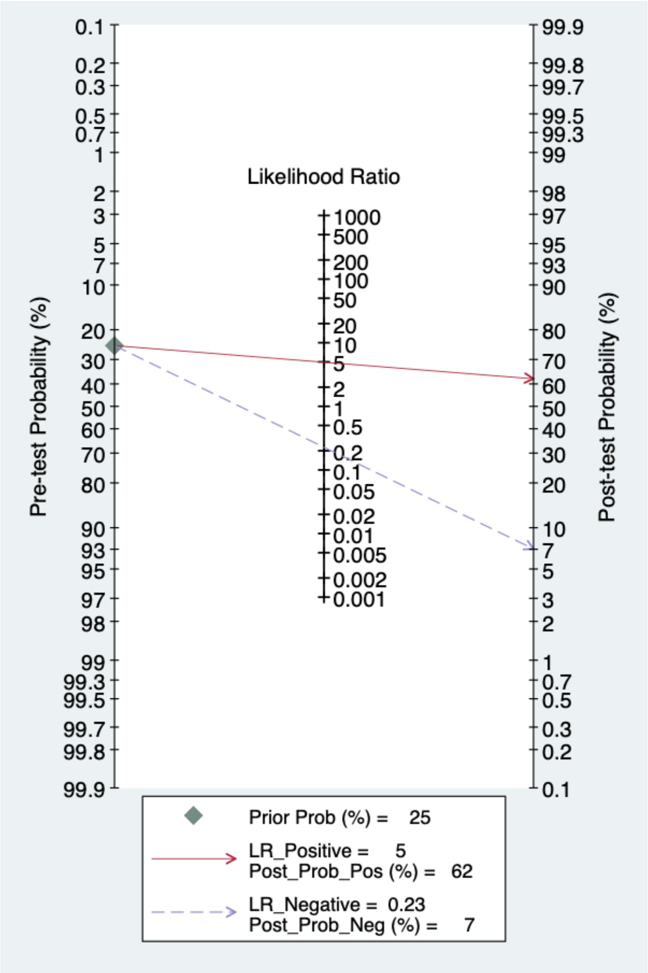

Supplementary Figure 4. Fagan plot analysis to evaluate the clinical utility of the machine learning method. The link between prevalence, probability ratio, and positive predictive value is depicted in this plot. The PPV indicates the probability that a patient with a positive test result actually has the condition. The likelihood that an individual with a negative test result is actually healthy is known as the negative predictive value, or NPV. The Fagan plot analysis indicated that the pre-test probability of machine learning was 25%, with subsequent probabilities following positive and negative results being 62% and 7%, respectively.

Supplementary 3. Single-arm Meta-analysis on Diagnostic Precision Properties

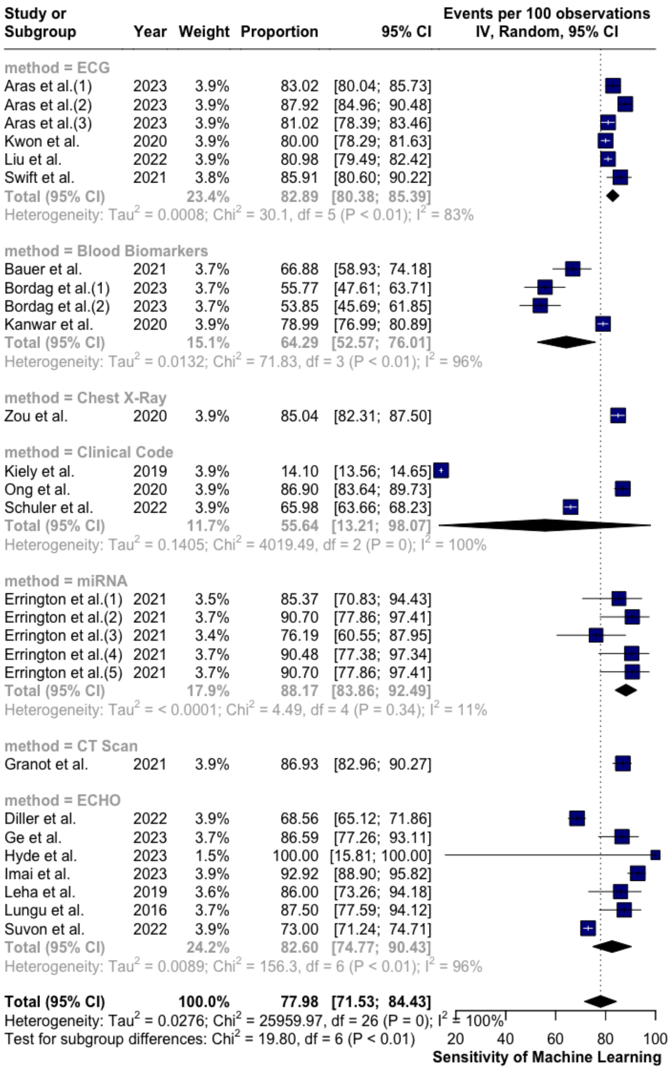

Supplementary Figure 5. Forest plot showing single-arm meta-analysis on sensitivity with corresponding heterogeneity statistics. The blue square and solid lines represent the odds ratio with 95% confidence intervals. The black rhombus indicates the pooled estimate with 95% confidence intervals.

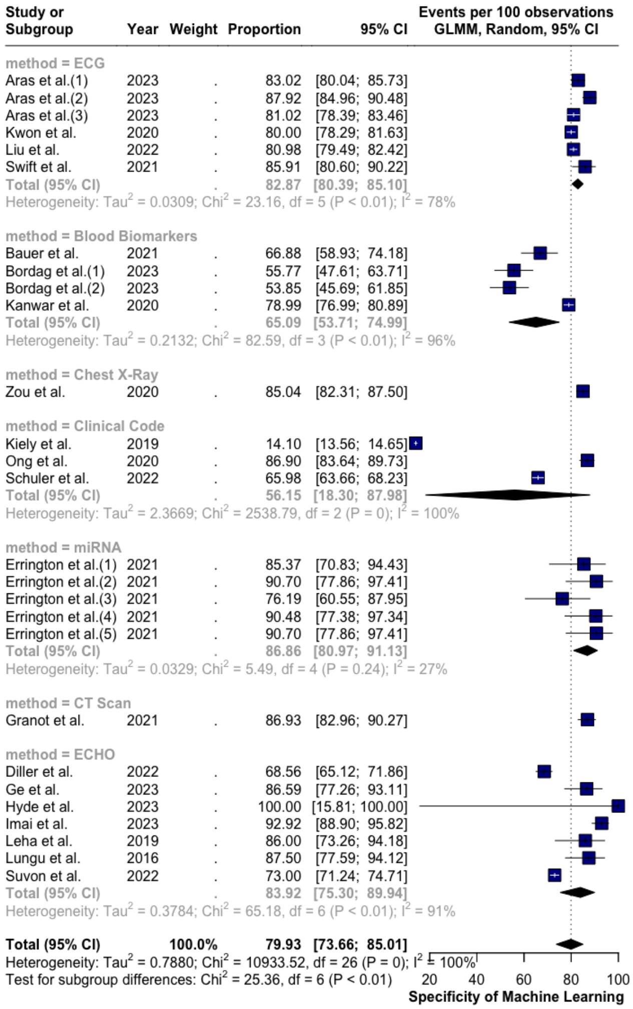

Supplementary Figure 6. Forest plot showing single-arm meta-analysis on specificity with corresponding heterogeneity statistics. The blue square and solid lines represent the odds ratio with 95% confidence intervals. The black rhombus indicates the pooled estimate with 95% confidence intervals.

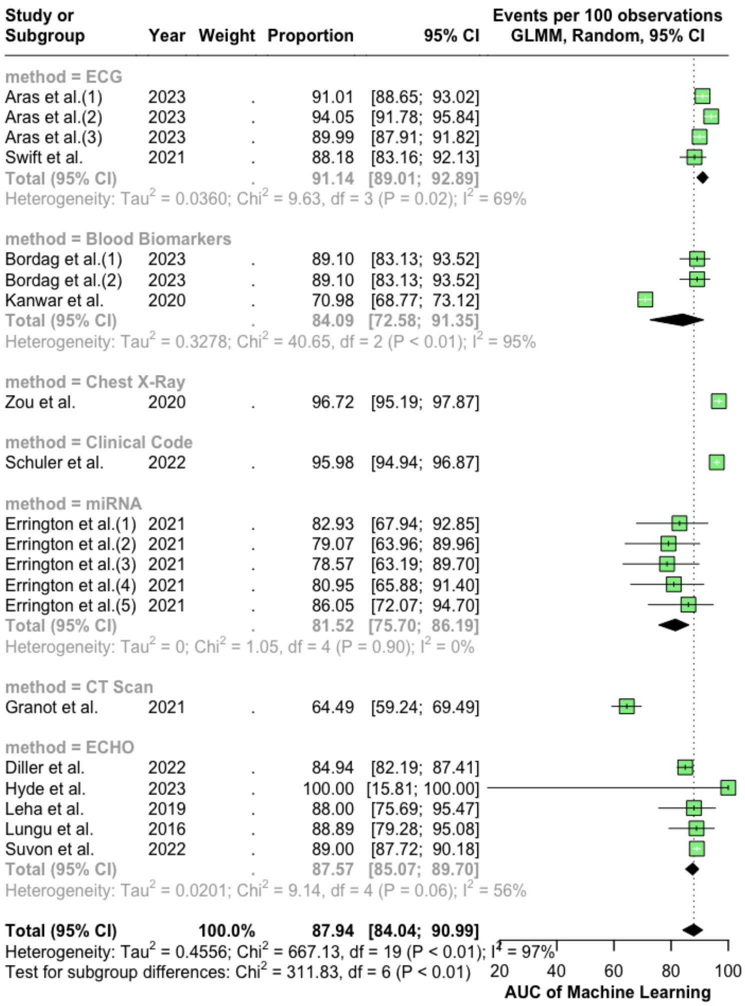

Supplementary Figure 7. Forest plot showing single-arm meta-analysis on the area under the curve with corresponding heterogeneity statistics. The green square and solid lines represent the odds ratio with 95% confidence intervals. The black rhombus indicates the pooled estimate with 95% confidence intervals.

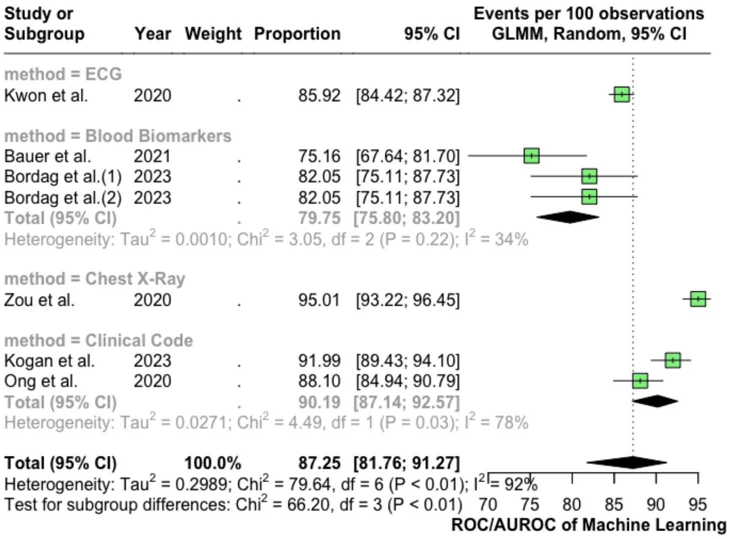

Supplementary Figure 8. Forest plot showing single-arm meta-analysis on the receiver operating characteristics with corresponding heterogeneity statistics. The green square and solid lines represent the odds ratio with 95% confidence intervals. The black rhombus indicates the pooled estimate with 95% confidence intervals.

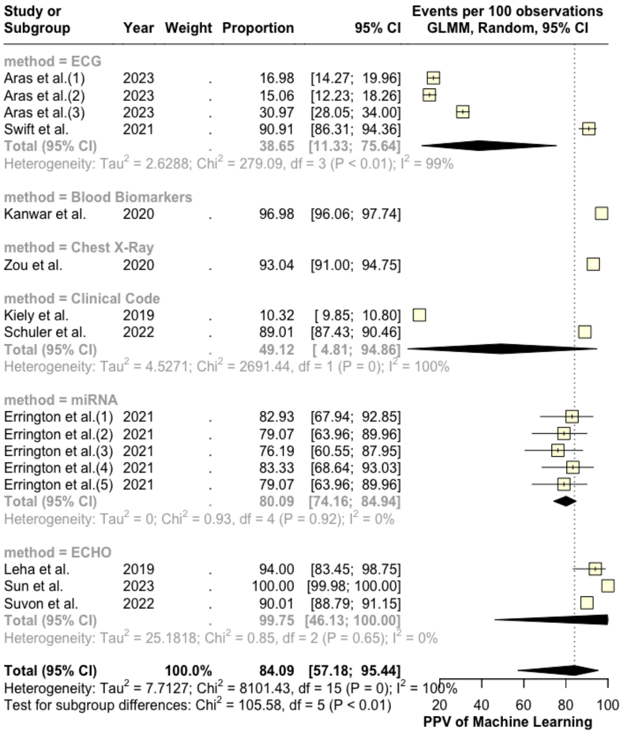

Supplementary Figure 9. Forest plot showing single-arm meta-analysis on positive predictive value with corresponding heterogeneity statistics. The yellow square and solid lines represent the odds ratio with 95% confidence intervals. The black rhombus indicates the pooled estimate with 95% confidence intervals.

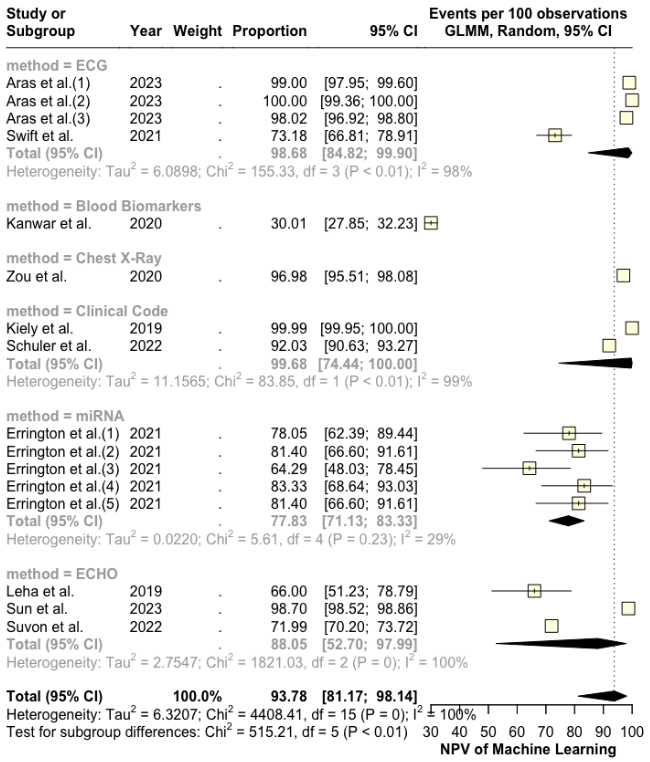

Supplementary Figure 10. Forest plot showing single-arm meta-analysis on negative predictive value with corresponding heterogeneity statistics. The yellow square and solid lines represent the odds ratio with 95% confidence intervals. The black rhombus indicates the pooled estimate with 95% confidence intervals.
